# Placental Inflammation in Preterm Premature Rupture of Membranes and Risk of Neurodevelopmental Disorders

**DOI:** 10.3390/cells14130965

**Published:** 2025-06-24

**Authors:** Elizabeth Marie Cervantes, Sylvie Girard

**Affiliations:** 1 Mayo Clinic Graduate School of Biomedical Sciences, Mayo Clinic, Rochester, MN 55905, USA; cervantes.elizabeth@mayo.edu; 2 Department of Obstetrics and Gynecology, Mayo Clinic, Rochester, MN 55905, USA; 3 Department of Immunology, Mayo Clinic, Rochester, MN 55905, USA

**Keywords:** preterm birth, neurodevelopmental disorders, placental inflammation, preterm premature rupture of membranes

## Abstract

Preterm premature rupture of membranes (pPROM) is a leading cause of preterm birth (PTB) and is increasingly recognized for its association with neurodevelopmental disorders (NDDs). The disruption of fetal membrane integrity introduces potential infection and inflammation into the intrauterine environment, triggering immune responses that may affect fetal development. Placental inflammation plays a pivotal role in mediating these effects, exposing the fetus to cytokines, oxidative stress, and potential microbial insults that contribute to adverse neurodevelopmental outcomes. This review examines the current evidence of the mechanistic pathways linking pPROM-induced placental inflammation to NDDs, emphasizing the roles of pathogen-associated molecular patterns (PAMPs) and damage-associated molecular patterns (DAMPs) in the inflammatory responses. We discuss how these immune activations lead to immune cell recruitment and excessive (or uncontrolled) production of inflammatory mediators, leading to an overall inflammatory imbalance that has been linked to disrupted fetal brain development in animal models. Animal models provide critical insights into how both sterile and pathogenic placental inflammation alter fetal neurodevelopment, while human studies, though limited, highlight promising biomarkers and potential therapeutic targets. This review identifies critical knowledge gaps and outlines future directions to mitigate the impact of placental inflammation on long-term infant health.

## 1. Introduction

Preterm birth (PTB) refers to birth occurring before 37 weeks of gestation, a critical cutoff for term pregnancy. PTB affects approximately 10% of pregnancies in the United States, with global rates ranging from 4% to 16% as of 2020 [1]. PTB remains the leading cause of neonatal morbidity and mortality worldwide [2].

PTB has several etiologies but approximately one third of all PTBs are due to preterm premature rupture of membranes (pPROM) [3]. pPROM is defined as the rupture of fetal membranes before 37 weeks of gestation and prior to the onset of labor. A key complication of pPROM is ascending infection, which leads to intrauterine inflammation, both of which are strongly linked to an increased risk of neurodevelopmental disorders (NDDs) [4].

NDDs encompass a wide range of adverse outcomes, from structural brain abnormalities (e.g., white matter injury, decrease in grey matter, decrease in cortical thickness) [5] to functional deficits (e.g., motor impairments, intellectual disabilities, and behavioral disorders) [6]. Examples of NDDs include cerebral palsy (CP), autism spectrum disorder (ASD), attention-deficit/hyperactivity disorder (ADHD) and learning disabilities, among others. The consequences of these impairments are profound, imposing lifelong social, financial, and emotional burdens on families. The impacts of NDDs are further exacerbated in low-resource settings and low socioeconomic status [7,8].

While PTB is an immediate and irreversible event, pPROM provides a critical latency period—spanning hours to weeks—between the rupture of membranes and delivery. This period provides a vital opportunity for targeted interventions aimed at reducing the burden on neonatal development, specifically leading to NDDs. Even in cases when prenatal intervention might not be possible, early identification of high-risk infants that may have experienced excess inflammation in utero is key to allow for postpartum clinical monitoring and early intervention.

PTB, particularly cases associated with pPROM, has been linked to a heightened risk of NDDs; these associations are thought to arise from in utero inflammation and its early disruptions of brain development [9]. Exposure to uncontrolled inflammation exacerbates the risk of altered brain development, as specific regions of the brain and different cell types, such as neurons and oligodendrocytes, are more vulnerable at various stages of development [10,11]. While prematurity itself is a known risk factor for NDDs due to incomplete brain development at birth, growing evidence suggests that uncontrolled inflammation—as seen in cases of pPROM—may play a central role in driving adverse neurodevelopmental outcomes [12,13,14].

The placenta and fetal membranes serve as a critical barrier between the maternal and fetal environments, regulating nutrient and oxygen exchange while providing a protective barrier against infections. pPROM disrupts this protective function, exposing the intrauterine environment to ascending pathogens and inflammatory mediators. Inflammation within the fetal membranes, including the chorion and amnion, contributes to a pro-inflammatory milieu that exacerbates placental dysfunction. Although fetal membrane inflammation plays a role mechanistically in the rupture of membranes, this review focuses primarily on the downstream effects of placental inflammation on fetal neurodevelopment [15,16].

While advancements in neonatal care have improved survival rates for preterm infants, the long-term neurodevelopmental outcomes following pPROM remain poorly understood, and early therapeutic strategies are lacking. This review aims to synthesize current knowledge on the relationship between pPROM, placental inflammation and neurodevelopmental outcomes. Placental inflammation disrupts cellular integrity, impairs nutrient exchange, and amplifies immune activation, further compounding fetal exposure to inflammatory insults [17,18,19,20]. By examining the pathways through which in utero inflammation alters fetal brain development, we highlight the critical need for early identification of at-risk infants and potential therapeutic strategies.

## 2. pPROM Etiology and Risk Factors

**Figure 1:**
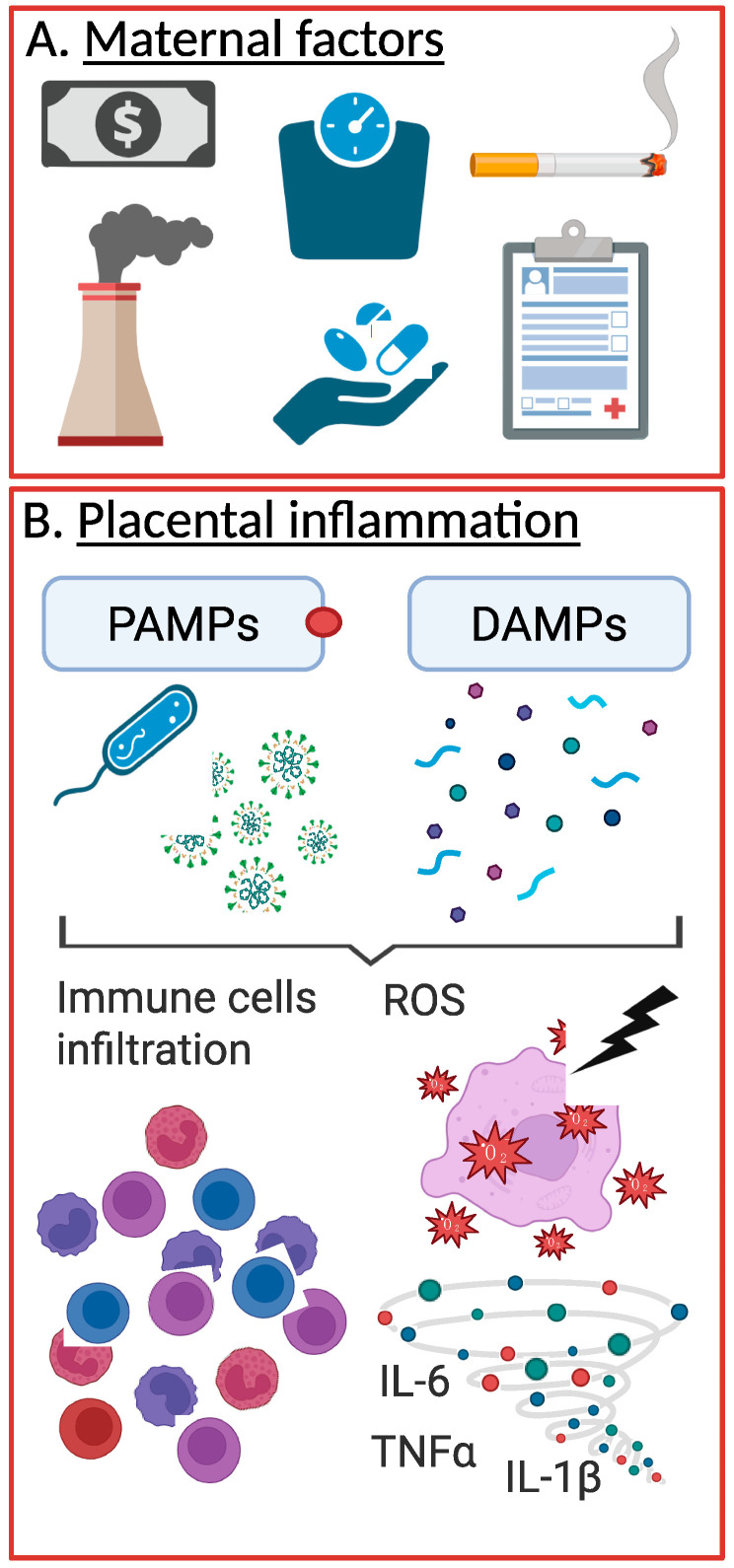
pPROM and placental inflammation. Several maternal factors have been linked to increased risk of pPROM, such as low income, elevated BMI, smoking, etc. (**A**). These factors, in addition to pPROM, are all causes of placental inflammation, either through infection, via PAMPs, or through sterile inducers of inflammation (i.e., DAMPs) (**B**). Created in Biorender.

The development of pPROM is influenced by a combination of maternal, fetal, and environmental risk factors. Maternal factors include a history of pPROM or PTB [21], which significantly increases recurrence risk, and conditions such as short cervical length [2], uterine overdistension [22] (e.g., from multiple gestations or polyhydramnios), and infections [22], each of which may increase the likelihood of pPROM. Lifestyle factors, such as low or high maternal body mass index (BMI), smoking, substance use, and low socioeconomic status, also play a role [21] (Figure 1A). Additionally, racial disparities are evident, with Black women being twice as likely as White women to experience pPROM and six times more likely to have recurrence [23]. These risk factors, often interrelated, underscore the complex etiology of pPROM.

Following the rupture of membranes, which is speculated to occur through several mechanisms, including microfractures, oxidative stress, premature senescence, infection, and apoptosis, among others, an inflammatory cascade is triggered [15]. This inflammation persists in maternal, fetal, and placental compartments, exacerbating the risk of adverse outcomes, including PTB and NDDs [24].

The developing fetal brain is highly susceptible to inflammatory insults, with earlier exposure leading to greater disruption of growth and differentiation; while prematurity itself increases the risk of NDDs, inflammation additionally exacerbates the risk [25,26]. Understanding the timing and impact of inflammation is essential for developing strategies to mitigate its effects and improve neurological outcomes for exposed infants.

In this section, we will discuss various complications and inflammatory processes that are observed as a result of pPROM and evidence that they may lead to impaired neurodevelopment.

### 2.1. Ascending Infections

Once pPROM occurs, the previously protected in utero environment is exposed to the non-sterile vagina, increasing the risk of ascending infection. The management of pPROM thus requires careful monitoring for signs of infection and the implementation of timely interventions, such as the administration of antibiotics and corticosteroids, to mitigate adverse outcomes whilst also minimizing the risk associated with early life exposure to these medications [3].

Vertical transmission of infection can occur while the fetus is in utero or passes as childbirth occurs. As a result, pathogens such as *Ureaplasma urealyticum*, *Mycoplasma hominis*, and Group B *Streptococcus* could ascend from the lower genital tract, disrupting membrane integrity through the infection and inflammation of the chorion, amnion, placenta and amniotic fluid, to varying degrees of severity [3,27,28,29].

While the specific mechanisms of infection and microbial contributions to pPROM have been extensively reviewed elsewhere [30,31,32], we will focus this review on the downstream impact of these infections—particularly placental inflammation—on neurodevelopmental outcomes, rather than the microbiological specifics. Inflammatory responses triggered by these infections play a critical role in mediating fetal injury, with downstream effects on brain development.

Given the placenta’s central role in nutrient exchange, immune signaling, and fetal brain development, our focus will be on how infection-related placental inflammation influences neurodevelopmental outcomes.

### 2.2. Causes of Placental Inflammation

The placenta is a temporary organ that is vital for fetal development, serving to nourish and protect the fetus through oxygen and nutrient delivery and as a barrier against microbial invasion and inflammation. Placental defenses against pathogens include physical barriers, such as the presence of a large multinucleated syncytiotrophoblast layer that lacks intercellular junctions, preventing pathogen movement, the secretion of antimicrobial effectors, and the activation of innate immune responses [33,34]. However, ascending infections can overcome these defenses, targeting critical structures such as the syncytiotrophoblast interface, the decidual–trophoblast interface, and other physical barriers.

Excessive inflammation of the placenta can compromise its function, reducing its capacity to facilitate nutrient and oxygen exchange and weakening its role as a physical barrier against inflammation. When its integrity is compromised, the risk of adverse neonatal outcomes increases significantly [35]. There are two main groups of inducers of placental inflammation, namely pathogen-associated molecular patterns (PAMPs) and damaged-associated molecular patterns (DAMPs), the latter also referred to as sterile mediators of inflammation. We will address each of these in detail below.

#### 2.2.1. Pathogen-Associated Inflammation

Pathogen-Associated Molecular Patterns (PAMPs) are molecular motifs derived from microorganisms, such as bacterial lipopolysaccharides (LPS), polyinosinic–polycytidylic acid (poly(I:C)), lipoteichoic acids, peptidoglycans, and nucleic acids [33] (Figure 1B). These structures are recognized by pattern recognition receptors (PRRs), including Toll-like receptors (TLRs) and NOD-like receptors (NLRs), expressed on placental cells such as trophoblasts, decidual cells, and macrophages [33]. The recognition of PAMPs by PRRs triggers a cascade of innate immune responses, activating signaling pathways that promote the release of pro-inflammatory cytokines and chemokines.

In the context of ascending infections, PAMPs shed by pathogens can directly interact with trophoblasts at critical interfaces, including the syncytiotrophoblast and decidual–trophoblast barriers [34]. This interaction compromises the placenta’s ability to maintain a sterile intrauterine environment and activates localized inflammation. The release of cytokines such as IL-1β, IL-6, IL-8, and TNF-α from infected trophoblasts and recruited immune cells amplifies the inflammatory response, potentially overwhelming the placenta’s capacity to limit damage. Comprehensive reviews on the innate immune mechanisms in response to infection in the placenta can be found here [1,33,34,36].

The placental inflammation associated with infections contributes to a pro-inflammatory intrauterine environment, which may, in turn, activate microglia—the brain’s resident immune cells. The potential mechanisms by which this inflammatory response impacts fetal neurodevelopment will be discussed in Section 3. While there are noted differences in severity between the types of infections via different microorganisms covered in depth here [28], all infections lead to placental inflammation and an inflamed in utero environment.

#### 2.2.2. Sterile Inflammation

While pathogen-driven inflammation follows a distinct trajectory, sterile inflammation presents unique challenges in the absence of active infection. Sterile inflammation refers to an inflammatory response triggered in the absence of an active infection, typically initiated by endogenous DAMPs released from injured or stressed cells. In the placenta, sterile inflammation can be driven by oxidative stress, cellular senescence, hypoxia, or mechanical stress, all of which may be more pronounced in pPROM [37]. Unlike PAMPs, DAMPs originate from host cells and include molecules such as uric acid, high-mobility group box 1 protein (HMGB1), ATP, cell-free fetal DNA and mitochondrial DNA [37] (Figure 1B). These molecules are typically found in low levels outside the cells, but when released in excessive quantities—as seen in the context of tissue injury and stress—they can perpetuate a harmful inflammatory cascade, contributing to cellular necrosis, placental dysfunction, and ultimately poor pregnancy outcomes [38].

Placental trophoblasts, macrophages, and other resident immune cells detect DAMPs via PRRs such as TLRs and NLRs. This detection triggers the release of pro-inflammatory cytokines and chemokines, including IL-1β, IL-6, and TNF-α, contributing to a localized inflammatory response. While this response aims to repair damage and maintain placental homeostasis, excessive or unresolved sterile inflammation can lead to significant placental dysfunction [39].

In the context of pPROM, sterile inflammation often arises from mechanical stress and oxidative damage caused by the loss of amniotic fluid and sustained disruption of the intrauterine environment. Oxidative stress results from an imbalance between reactive oxygen species (ROS) production and the placenta’s antioxidant defense, damaging cellular components, such as lipids, proteins, and DNA, further exacerbating the release of DAMPs and perpetuating inflammation [40]. Under normal conditions, the placenta employs a robust antioxidant system to neutralize ROS and protect fetal tissues. However, in pPROM, the prolonged loss of amniotic fluid and increased exposure to inflammatory stimuli overwhelm these protective mechanisms, leading to excessive ROS accumulation [40]. This stress damages cellular components, such as lipids, proteins, and DNA, further exacerbating the release of DAMPs and perpetuating inflammation.

In parallel, cellular senescence in trophoblasts—key placental cells responsible for nutrient exchange and hormone production—exacerbates inflammation through the secretion of pro-inflammatory mediators known as the senescence-associated secretory phenotype (SASP) [41]. Senescent trophoblasts release cytokines (e.g., IL-6, IL-8, and TNF-α), chemokines, and matrix metalloproteinases, which not only sustain a chronic inflammatory environment but also contribute to extracellular matrix degradation and placental insufficiency [42,43,44]. This can disrupt trophoblast function, impair nutrient and oxygen exchange, and compromise the structural integrity of the membranes [37,38,45].

The combined effect of oxidative stress and SASP amplifies an inflammatory placental environment that is toxic to the fetus [44]. Chronic exposure to this environment could disrupt fetal brain development through multiple pathways. Excessive inflammation and oxidative stress, as demonstrated in animal models, trigger increased astrogliosis and microgliosis in the white matter, alter myelination, and lead to behavioral differences in offspring exposed to sterile inflammation compared to controls [46]. Moreover, persistent oxidative and inflammatory stress in utero can lead to epigenetic modifications, such as DNA methylation and histone modifications, that may alter gene expression patterns crucial for brain development [47,48]. These epigenetic changes can have lasting consequences, affecting neurodevelopmental trajectories well beyond the perinatal period.

Current research suggests that sterile inflammation is a critical yet underexplored mechanism in placental pathophysiology. Understanding its role in conditions like pPROM could inform therapeutic strategies aimed at mitigating oxidative stress and resolving inflammation, potentially improving outcomes for infants.

#### 2.2.3. Pathogen-Driven and Sterile Inflammation in pPROM: Acute and Chronic Inflammatory Response

Regardless of the cause, pathogenic (PAMPs) or sterile (DAMPs), inflammation of the placenta and its surrounding tissues is characterized by both the type of immune cell infiltration (acute vs. chronic) and its origin (maternal vs. fetal). Acute inflammation is marked by the infiltration of neutrophils, which rapidly respond to infection or injury. In the context of pPROM, acute inflammation often presents as clinical chorioamnionitis, an acute inflammation of the fetal membranes typically associated with infection. Chorioamnionitis is diagnosed based on clinical signs, including maternal fever, maternal tachycardia, fetal tachycardia, and foul-smelling amniotic fluid [49]. Post-delivery, the placenta can undergo pathological evaluation to identify signs of histological chorioamnionitis or microbiological testing to culture pathogens from the chorioamnion. However, studies examining the association of chorioamnionitis and neonatal outcomes and further blinded epidemiological and mechanistic studies are essential to determine the precise impact of chorioamnionitis on neonatal and childhood outcomes [50,51].

In contrast, chronic placental inflammation involves sustained immune responses characterized by the infiltration of immune cells other than neutrophils, such as maternal T cells and macrophages. Cytokines and chemokines released by placental macrophages and trophoblasts stimulate an inflammatory response that can attract maternal immune cells. Examples of chronic placental inflammation include villitis, with the invasion of maternal T cells into the placental villi, causing necrosis, sclerosis and fibrosis [52]. Other chronic conditions include chronic chorioamnionitis and chronic deciduitis [24]. A better understanding of these distinct inflammatory pathways may inform targeted therapeutic interventions aimed at mitigating the adverse effects of both acute and chronic inflammation in cases of pPROM.

## 3. Placental Inflammation and Neurodevelopmental Disruptions

pPROM has been attributed to various biological mechanisms within the placenta, as explained above, which all contribute to fetal exposure to an inflammatory environment [9,53,54]. These uncontrolled inflammatory processes not only reflect the intrauterine environment’s response to membrane rupture but also contribute to the cascade of complications that can influence neonatal outcomes and alter the long-term developmental trajectories.

Given the placenta’s pivotal role in fetal development, inflammation-induced dysfunction extends its impact to the developing brain. While much remains to be understood in humans, recent studies—using both animal models and human data—have begun to shed light on the mechanism by which placental inflammation may influence neurodevelopment.

### 3.1. Animal Models of Prenatal Inflammation’s Impact on Neurodevelopment

Animal studies have extensively examined the effect of prenatal inflammation on brain injury, particularly through placental inflammation and the inflammatory milieu created in response to inducers of pathogenic or sterile origin. Many studies have highlighted the potential mechanisms through which inflammation disrupts fetal brain development, including microglial activation, cytokine-mediated neurotoxicity, oxidative stress, and sex-specific vulnerabilities [15,55,56,57].

**Figure 2:**
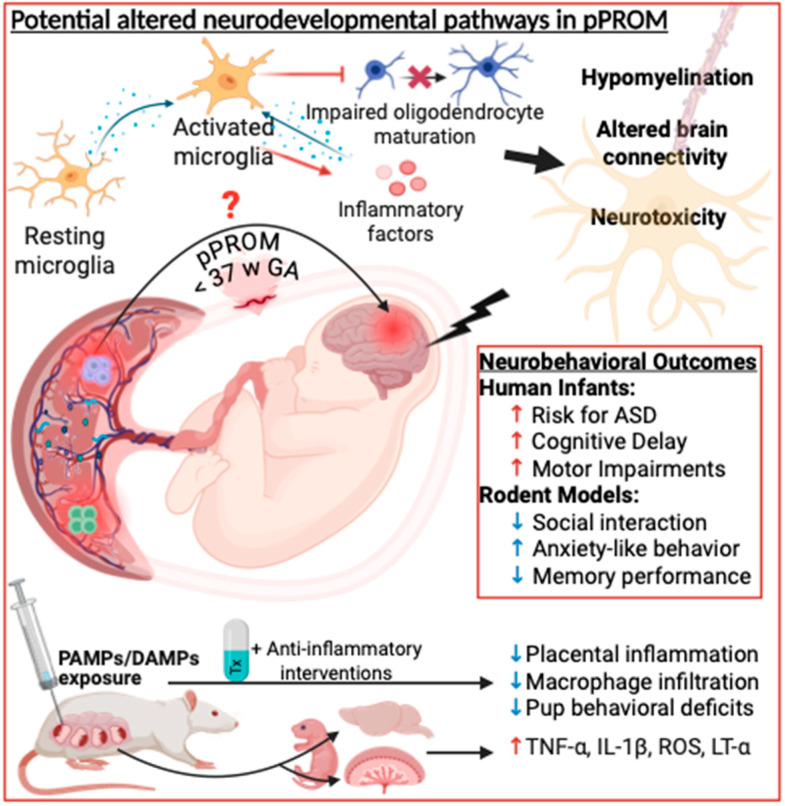
Link between pPROM, placental inflammation and neurodevelopmental disorders. Multiple studies have shown the link between prenatal inflammation and NDD. This causal link has been shown using animal models and the association reported in human studies with the potential links and therapeutic intervention used in animal models, shown in the figure. Created in Biorender.

One key mechanism of inflammation-induced brain injury is through the activation of microglia. There have been conflicting reports regarding whether maternal inflammatory mediators (e.g., cytokines) can cross the placental barrier, or if these can be produced on the fetal side of the placenta and reach the premature brain through the blood–brain barrier [14,58,59]. Cytokines contribute to microglial activation, which leads to neuronal death and an increase in extracellular adenosine triphosphate concentration, further amplifying local inflammation [60] (Figure 2). Interestingly, in a rat model of prenatal LPS administration, leading to placental damage, maternal cytokines did not reach the fetus, even though direct placental production on the fetal side of the placenta cannot be ruled out [59]. When primed, microglia can also release inflammatory mediators such as TNF-α, IL-1β, and ROS, which can further impair neurons and sustain a cycle of neuroinflammation and degeneration [61]. Additionally, TNF-α, whether by crossing the BBB or directly excreted from activated microglia, has been shown to be cytotoxic in neural tissue and increased in brain tissue following LPS stimulation [62,63].

Prenatal exposure to LPS has been shown to lead to neurodevelopmental alterations, both at the structural and functional levels in rodents, including behavioral defects reminiscent of what is observed in infants with neurodevelopmental disorders [64,65,66,67] (Figure 2. Of note is that the precise timing of the exposure to inflammation is important and defines the brain alteration that will ensue [68,69]. In mouse models, poly(I:C) exposure during mid-pregnancy leads to deficits in social behavior, communication and motor skills in the pups through elevated cytokines in the placenta [70]. Of high interest is that a key study highlighted sex-specific differences in inflammatory responses and neurodevelopmental outcomes following prenatal immune activation using poly(I:C). Their analyses revealed that male offspring exhibited elevated placental levels of pro-inflammatory cytokines such as IL-6, TNFα, and LT-α, whereas female offspring did not show these increases [71]. However, other studies have observed changes in behavior in both sexes [72], although the exact mechanisms involved might be unique to each sex [71].

Studies in rats have shown that the negative impact of prenatal inflammation was mediated by the impact on the placenta and that prenatal anti-inflammatory treatment targeting the IL-1 system was beneficial against neurodevelopmental alterations, only when placental protection was achieved [73,74,75]. Early detection of inflammation through magnetic resonance imaging, which identified increased water content and tissue changes associated with inflammatory processes, allowed for the timely administration of IL-1Ra, which protected the placenta by preserving tissue integrity and limiting macrophage infiltration [76]. Protection by IL-1Ra was also observed in a model of prenatal exposure to sterile inflammation through uric acid [46]. Other work targeting the IL-1 system using an allosteric antagonist of IL-1, rytvela, effectively reduced PTBs and improve neonatal outcomes by suppressing inflammation in offspring of mice exposed to LPS [77,78]. In addition, several models of live infections (i.e., GBS, E. Coli, influenza, Zika, etc.) during pregnancy have shown the impact on the pups’ neurodevelopment [57,79,80,81].

Targeting other inflammatory pathways, such as anti-TNF-α, has shown conflicting evidence of efficacy, and has been shown to be associated with adverse pregnancy outcomes [82]. For instance, research has demonstrated that TNF-α antagonists can attenuate systemic lipopolysaccharide-induced brain white matter injury in neonatal rats, suggesting potential neuroprotective effects [83]. However, others have reported that anti-TNF-α drugs, such as infliximab and etanercept, are associated with adverse pregnancy outcomes, including intrauterine growth restriction, spontaneous abortion, and PTB [82,84]. Tocilizumab, an anti-IL-6 receptor monoclonal antibody, has shown promise in reducing inflammation associated with PTB. In preclinical models, it demonstrated the ability to reduce the inflammatory cytokine levels and protect against uterine activation, improving fetal outcomes by preventing fetal mortality and PTB [85].

Of interest is that other anti-inflammatory drugs during pregnancy, such as meloxicam, which acts through COX-1/2 inhibition, reduced some inflammatory markers in the offspring, but did not fully prevent the structural and behavioral deficits induced by prenatal LPS exposure [64]. This suggests that while anti-inflammatory interventions may mitigate certain aspects of prenatal inflammation, a better understanding of their actions, including the timing of administration and site of action, is needed to achieve therapeutic potential.

#### Summary and Future Directions in Animal Models

These studies collectively illustrate how placental inflammation acts as a key driver of fetal brain injury through multiple interconnected mechanisms. Pro-inflammatory cytokines such as IL-1, IL-6 and TNF-α are key inflammatory mediators in the placenta and are associated with abnormal neurodevelopment. Animal models have demonstrated that exposure to inflammation in utero results in long-term structural and functional impairments, and cognitive and behavioral deficits. Additionally, sex-specific differences have emerged, with male fetuses exhibiting heightened inflammatory responses and greater neurodevelopmental vulnerability [86].

While some treatments, such as meloxicam or IL-1Ra, in animal models have shown partial attenuation in reducing inflammatory markers, they do not fully prevent neurodevelopmental impairments, highlighting differences in altered neuroanatomy and neurotransmitter levels after administration of the drug. This underscores the need for more effective therapeutic strategies aimed at mitigating the lasting impact of prenatal inflammation on brain development. The extent of brain injury appears to depend on the timing, duration, and severity of the inflammatory exposure, with earlier gestational insults leading to more profound neurological consequences. While existing studies provide critical insights into these pathways, further research is necessary to delineate precise mechanisms and identify potential therapeutic targets to mitigate the long-term consequences of prenatal inflammation. These animal model findings align with emerging clinical observations in human studies, offering potential mechanistic links and shared inflammatory pathways. However, notable differences in timing, immune responses, and potential compensatory mechanisms between animal models and human pregnancies underscore the importance of careful translational interpretation. In the next section, we compare these experimental findings to human studies to highlight converging evidence and key translational gaps.

### 3.2. Human Studies

While animal models provide significant insights into the mechanistic pathways by which placental inflammation contributes to neurodevelopmental disruptions, comparing these findings with human studies is essential to strengthen the evidence base and refine our understanding of clinical implications. Direct evidence in humans remains limited, but parallels with animal studies offer important translational insights. In such cases, longitudinal and observational studies, as well as parallels from other conditions where placental pathology informs neurodevelopmental risk, can provide critical insight.

A retrospective cohort study examined the relationship between placental inflammatory cytokine mRNA expression and cognitive performance in preschool-aged children [53]. The study found that increased placental expression of pro-inflammatory cytokines, IL-6 and TNF-α, was associated with lower cognitive performance. Elevated levels of these cytokines, which play key roles in the fetal inflammatory response, were linked to deficits in memory, attention, and overall cognitive function [53]. These findings suggest that elevated placental inflammation may adversely affect early childhood cognitive development. This aligns with animal findings where placental pro-inflammatory cytokines were linked to neurodevelopmental deficits, suggesting conserved pathways across species. These parallels between animal and human findings reinforce the translational significance of targeting placental inflammation as a modifiable factor in neurodevelopmental risk.

This connection between placental inflammation and neurodevelopment is further explored through studies on maternal infections, such as SARS-CoV-2 infection. One study examined the impact of infection on placental macrophages (Hofbauer cells (HBCs)) and their potential role in neurodevelopment [87]. The researchers identified subpopulations of HBCs with altered gene expression profiles, suggesting impaired phagocytic function and an inflammatory phenotype. These changes may disrupt the placental microenvironment, potentially influencing fetal brain development by altering pro- and anti-inflammatory signals that regulate fetal microglial maturation. The study also explored how these altered HBCs affected microglial-like cells derived from human pluripotent stem cells, observing shifts in gene expression and cell function following exposure. However, the relevance to actual microglia in the developing brain remains uncertain [87]. This study highlights a potential link between maternal SARS-CoV-2 infection and fetal brain development, but further research is needed to determine whether similar effects occur in real microglial cells. Maternal SARS-CoV-2 infection studies highlight the role of placental macrophages in shaping neurodevelopmental risk, consistent with animal studies of viral infection showing that inflammatory placental responses contribute to microglial activation and later brain injury [88].

Zika virus infection during pregnancy has been strongly linked to severe neurodevelopmental disorders, including microcephaly and other congenital brain abnormalities, due to its ability to cross the placenta and directly infect neural progenitor cells [89,90,91,92]. Similarly, maternal influenza infection—especially during the first trimester—has been associated with increased risk of neurodevelopmental disorders such as autism spectrum disorder and schizophrenia, likely mediated by systemic maternal immune activation and elevated pro-inflammatory cytokines [93]. These infection studies in humans also mirror findings from animal models where direct or indirect placental inflammation impacts fetal brain development.

The importance of placental health in neurodevelopment is further illustrated by research in specific populations, such as neonates with congenital heart disease (CHD) [94,95]. Placental lesions are associated with impaired volumetric brain development in neonates with CHD, as well as adverse neurodevelopmental outcomes later in life. These findings underscore the crucial role placental health plays in shaping fetal brain development, even in the context of other complex medical conditions. While much of the existing research has focused on specific conditions, broader studies have also explored the association between placental pathology and neurodevelopmental outcomes. Literature has emphasized that placental lesions, such as infarct and chronic inflammation, can impact fetal development and predict neurodevelopmental delays, potentially serving as early indicators of later cognitive and motor deficits [96,97,98,99]. These studies highlight the importance of integrating placental pathology into our understanding of fetal development and its long-term impacts on neurodevelopment.

Taken together, these human studies not only corroborate key findings from animal models but also underscore the importance of integrated research efforts. Bridging these approaches will be vital for translating mechanistic understanding into early biomarkers and intervention strategies to reduce the burden of inflammation-mediated neurodevelopmental delays. Given the increasing evidence linking placental inflammation to neurodevelopmental outcomes, further exploration in this area is crucial. Future research should focus on identifying specific biomarkers associated with placental lesions that could predict neurodevelopmental outcomes. This would enable the development of targeted monitoring strategies during pregnancy, such as tracking inflammatory markers (e.g., cytokines, placental damage) to assess fetal risk. Additionally, early interventions could include therapeutic strategies to reduce placental inflammation or postnatal approaches aimed at mitigating neurodevelopmental delays. By exploring the mechanistic pathways linking placental pathology to brain development, we can develop more effective strategies to reduce both placental inflammation and subsequent impact on the fetus, which may be linked to NDDs.

## 4. Perspectives

Looking ahead, understanding the impact of placental inflammation on childhood neurodevelopment is critical for improving outcomes in high-risk pregnancies. Neonates exposed to placental inflammation are at an increased risk for long-term developmental challenges, including cognitive delays, behavioral disorders, and psychiatric conditions. Early identification of infants at risk, through biomarkers and placental pathology analysis, can help guide targeted interventions, such as neuroprotective therapies and developmental monitoring.

Within this broader context, pPROM represents a particularly important model of inflammation-associated risk. As both a potential result of intrauterine inflammation and a condition that exacerbates fetal exposure to inflammatory stimuli, pPROM uniquely situates the fetus in a high-risk environment for disrupted neurodevelopment. Furthermore, it is not yet clear whether fetal brain injury occurs before or after membrane rupture, nor whether placental inflammation initiates or is a consequence of pPROM. Future work should prioritize pPROM-specific cohorts to investigate whether early identification of placental or fetal biomarkers after membrane rupture can help predict neurodevelopmental outcomes and guide clinical follow-up. Moreover, placental inflammation is also a hallmark of pregnancy complications such as preeclampsia and fetal growth restriction, which increases the risk of neurodevelopmental impairments. These observations raise the possibility of shared pathways across these pregnancy complications even though distinct profiles were recently shown [100]. Direct comparative studies are sparse and needed.

Integrating molecular profiling of the placenta with non-invasive prenatal testing could enhance early detection efforts and inform timely interventions. To establish causal relationships, in vivo models, such as those utilizing rodents or other animals, are crucial for directly observing the impact of placental inflammation on fetal neurodevelopment. In humans, further epidemiologic studies with rigorous longitudinal follow-up will be crucial to clarify these associations and to identify early biomarkers of neurodevelopmental risk—potentially leveraging placental, cord blood, and other perinatal biospecimens for predictive value. In animal models, the synthesized findings in this review can inform mechanistic studies that dissect the precise timing, causality, and shared inflammatory pathways underlying these complications. Together, these translational approaches will be key for ultimately improving neurodevelopmental outcomes in infants exposed to pPROM-related inflammation. Future directions should focus on translating these findings into actionable clinical practices. Early developmental intervention programs demonstrate potential for improving motor and cognitive outcomes in preterm infants. However, the evidence also suggests that the timing of these interventions—whether implemented in infancy, preschool, or school age—can yield varying results in physical and mental outcomes [101]. Recognizing these nuances highlights the need for individualized approaches that consider both timing and intensity of intervention. Incorporating biomarkers of prenatal inflammation and early-life adversity into care plans may further refine these efforts, guiding targeted strategies to enhance neurodevelopmental outcomes.

Clinically, targeting placental inflammation holds promise for improving neonatal neurodevelopment. Although preclinical data support interventions like IL-1Ra and tocilizumab, some of these agents have not been tested in pregnant humans. IL-1 blockers in general, such as IL-1Ra, used for other indications and continued during pregnancy have been reported to be safe during pregnancy, as shown in a recent systematic review [102].

## 5. Conclusions

In summary, there is clear evidence from both preclinical models and human studies of the association between pPROM/placental inflammation and neurodevelopmental disorders. The translation of these research findings into clinical practice could ultimately lead to new screening protocols, precision medicine approaches, and targeted anti-inflammatory therapies to mitigate the adverse effects of prenatal inflammation on fetal brain development. Personalized approaches that consider the timing, severity, and sex-specific responses to inflammation are needed, especially for conditions like pPROM, where gestational age at rupture and infection status may further shape outcomes. By continuing to explore these pathways, researchers and clinicians can work toward reducing the burden of neurodevelopmental disorders and improving lifelong health outcomes for affected individuals.

## Data Availability

Not applicable.
